# Structure-Based Virtual Screening Reveals Ibrutinib and Zanubrutinib as Potential Repurposed Drugs against COVID-19

**DOI:** 10.3390/ijms22137071

**Published:** 2021-06-30

**Authors:** Satyavani Kaliamurthi, Gurudeeban Selvaraj, Chandrabose Selvaraj, Sanjeev Kumar Singh, Dong-Qing Wei, Gilles H. Peslherbe

**Affiliations:** 1 Centre for Research in Molecular Modeling & Department of Chemistry and Biochemistry, Concordia University, Montreal, QC H3G 1M8, Canada; satyavani.mkk@gmail.com (S.K.); gurudeeban.selvaraj@concordia.ca (G.S.); 2 Computer Aided Drug Design and Molecular Modeling Lab, Department of Bioinformatics, Alagappa University, Karaikudi 630003, India; selnikraj@bioclues.org (C.S.); sksingh@alagappauniversity.ac.in (S.K.S.); 3 The State Key Laboratory of Microbial Metabolism, College of Life Sciences and Biotechnology, Shanghai Jiao Tong University, Shanghai 200240, China

**Keywords:** protein-ligand binding free energy, BTK inhibitors, COVID-19, MD simulations, SARS-CoV-2, ibrutinib, zanubrutinib

## Abstract

Coronavirus disease (COVID)-19 is the leading global health threat to date caused by a severe acute respiratory syndrome coronavirus (SARS-CoV-2). Recent clinical trials reported that the use of Bruton’s tyrosine kinase (BTK) inhibitors to treat COVID-19 patients could reduce dyspnea and hypoxia, thromboinflammation, hypercoagulability and improve oxygenation. However, the mechanism of action remains unclear. Thus, this study employs structure-based virtual screening (SBVS) to repurpose BTK inhibitors acalabrutinib, dasatinib, evobrutinib, fostamatinib, ibrutinib, inositol 1,3,4,5-tetrakisphosphate, spebrutinib, XL418 and zanubrutinib against SARS-CoV-2. Molecular docking is conducted with BTK inhibitors against structural and nonstructural proteins of SARS-CoV-2 and host targets (ACE2, TMPRSS2 and BTK). Molecular mechanics-generalized Born surface area (MM/GBSA) calculations and molecular dynamics (MD) simulations are then carried out on the selected complexes with high binding energy. Ibrutinib and zanubrutinib are found to be the most potent of the drugs screened based on the results of computational studies. Results further show that ibrutinib and zanubrutinib could exploit different mechanisms at the viral entry and replication stage and could be repurposed as potential inhibitors of SARS-CoV-2 pathogenesis.

## 1. Introduction

Coronavirus disease (COVID)-19 is the leading global health threat to date caused by a coronavirus (SARS-CoV-2 or nCov-2019) next to severe acute respiratory syndrome (SARS) and the Middle East respiratory syndrome (MERS) [1,2] in this century. Though antiviral and antimalarial drugs are being used to control and enhance the recovery rate from COVID-19, the death toll rate is still increasing. There are few vaccine candidates prescribed by the World Health Organization, including BNT162b2, mRNA-1273, Covishield, Sputnik V, JNJ-78436735, CoronaVac, Sinovac and Covaxin, for emergency use by frontline workers and the general public to create herd immunity. However, there is no unique or effective medication available for COVID-19. Nearly 25 million people had been affected and 800 thousand lives had been lost due to the COVID-19 pandemic as of March 2021 [3].

Recent clinical studies have raised the question of the possible use of Bruton’s tyrosine kinase (BTK) inhibitors (i.e., acalabrutinib, ibrutinib, dasatinib and zanubrutinib) to treat COVID-19 patients, and, surprisingly, it resulted in improved oxygenation in the majority of patients with no discernable toxicity [4], reduced SARS-CoV-2 symptoms (i.e., dyspnea and hypoxia) [5] and reduced thromboinflammation and hypercoagulability [6,7]. Bruton’s tyrosine kinase (BTK) is a non-receptor tyrosine kinase; it plays a pivotal role in the development, maturation, differentiation and proliferation of B lymphocytes (B cells) [8]. Initially, BTK was identified as a potential mediator of B cell receptor-mediated signaling, especially in the activation of adaptive immunity. The abnormal level of inflammatory responses by the cytokine storm leads to severe acute respiratory distress syndrome (ARDS); it is considered a significant hallmark of SARS-CoV-2 [9]. An elevated level of cytokines (IL-1β, IFNγ and TNF-α) has been recorded in COVID-19 patients who are admitted to intensive care and non-intensive care units as compared to healthy adults [10]. A recent scientific article from the Howard Hughes Medical Institute on 21 May 2020 showed that preventing the cytokine storm may reduce the severity of COVID-19 [11].

Increased evidence of BTK’s active participation in macrophage signaling and activation, sensing invading pathogens through various toll-like receptors (TLRs) and innate immunity has been reported [4,12,13]. TLRs on macrophages recognize the single-stranded genomic RNA of viruses similar to SARS-CoV-2 and initiate the BTK-dependent activation of the nuclear factor kappa-light-chain-enhancer of activated B cells (NF-κB) signaling pathway which promotes the secretion of various inflammatory cytokines against pathogens [14,15]. However, the dysregulated BTK signaling pathway in lung macrophages may be a key pathophysiological component of SARS-CoV-2-related lung injury [5]. In general, cytokines are clusters of proteins that include chemokines, interferons (IFNs), interleukins (ILs), lymphokines and tumor necrosis factors produced by immune cells against pathogenic substances. Earlier, Phase I/II clinical trials showed the safety and efficacy profile of BTK inhibitors (ibrutinib and acalabrutinib) in patients with refractory mantle cell lymphoma and chronic lymphocytic leukemia [16,17]. However, long-term BTK inhibitor therapies feature common adverse events with mild-to-moderate signs such as diarrhea (off-target adverse effect related to EGFR inhibition), pyrexia, fatigue, constipation, pneumonia and upper respiratory tract infections in very rare cases [18,19]. However, detailed experimental findings may be needed to confirm the action and adverse effects of BTK inhibitors in a large number of clinical trials. Owing to the COVID-19 pandemic, there is an imperative need for drug repurposing towards dual intervention with BTK-mediated cytokine storm and viral protease complex but with fewer side effects.

**Figure 1:**
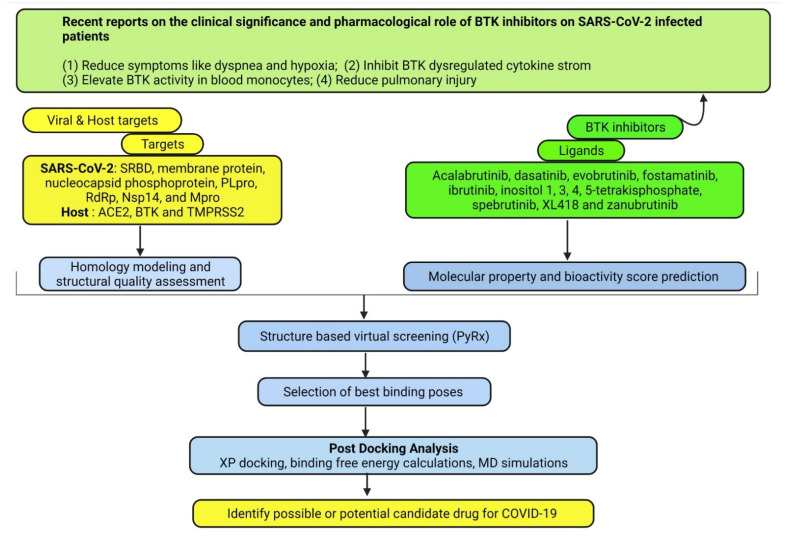
Overview and workflow of this study. ACE2—angiotensin-converting enzyme 2; BTK—Bruton’s tyrosine kinase; MD—molecular dynamics; Mpro—main protease; nsp—nonstructural protein; PLpro—papain-like protease; RdRp—RNA-dependent RNA polymerase (RdRp), SRBD—spike receptor-binding domain; TMPRSS2—transmembrane serine protease 2.

Drug development from scratch (lead identification and synthesis, pre-clinical and clinical research, FDA review, postmarket and drug safety monitoring) is a lengthy process which may not be the best suited to yield immediate solutions to the COVID-19 pandemic and related public health issues. On the other hand, drug repurposing is one of the effective emerging strategies to control COVID-19. During this pandemic situation, in silico approaches such as structure-based virtual screening (SBVS) and molecular dynamics (MD) simulations can be critical in identifying antiviral inhibitors against the dreadful COVID-19 [20,21,22,23]. Figure 1 illustrates the flow of the study: acalabrutinib, dasatinib, evobrutinib, fostamatinib, ibrutinib, inositol-tetrakisphosphate, spebrutinib, XL418 and zanubrutinib were selected as ligands and SARS-CoV-2 structural proteins including the spike receptor-binding domain (SRBD), the membrane protein, the nucleocapsid phosphoprotein, and nonstructural proteins (nsp) such as RNA-dependent RNA polymerase (RdRp), Nsp14, main protease (Mpro) and papain-like protease (PLpro) were selected as targets. The spike glycoprotein of SARS-CoV-2 binds with the host cell surface receptor angiotensin-converting enzyme 2 (ACE2), whereas transmembrane serine protease 2 (TMPRSS2) activates its membrane fusion. In addition, recent studies reported that BTK signaling dysregulation in lung macrophages might be a key pathophysiological component of SARS-CoV-2-induced lung injury [24,25]. Thus, ACE2, TMPRSS2 and BTK were also selected as drug targets for COVID-19. However, no computational study has been reported on the potential of BTK inhibitors against SARS-CoV-2. Therefore, integrated computational analysis (SBVS, MM/GBSA and MD simulations) was performed using nine ligands with seven viral targets and three host targets to find out possible candidate drugs against COVID-19.

## 2. Results

### 2.1. Molecular Property and Bioactivity Score Prediction

Table 1 collects the molecular properties and bioactivity scores of the selected drugs (acalabrutinib, dasatinib, evobrutinib, fostamatinib, ibrutinib, inositol-tetrakisphosphate, spebrutinib, XL418 and zanubrutinib). According to Lipinski’s rule of five, ideal drug molecules fall under such criteria as logP (≤ 5), molecular weight (< 500 daltons), number of hydrogen acceptors (≤ 10), number of hydrogen donors (≤ 5) and number of atoms (from 20 to 70) [26]. The octanol–water partition coefficient (logP) values reflect the compound’s hydrophobicity, mostly employed in quantitative structure–activity relationship (QSAR) studies. Drugs with 10 or fewer rotatable bonds feature good prospects of oral bioavailability.

Acalabrutinib, dasatinib, evobrutinib, ibrutinib, XL418 and zanubrutinib satisfy Lipinski’s rule of five [molecular weight (< 500 g/mol), logP values (range from 2.26 to 4.50), rotatable bonds (≤ 10), HB acceptors (< 10), number of HB donors (< 5) and number of atoms (> 30)]. Fostamatinib, inositol-tetrakisphosphate and spebrutinib violate Lipinski’s rule. The total polar surface area (TPSA) reflects the permeability or bioavailability of compounds. The selected ligands showed lower TPSA values (ranging from 97.40 to 118.52 Å^2^), excluding fostamatinib and inositol-tetrakisphosphate; the ligands that possess a TPSA value below 150 Å^2^ have good permeability. Various bioactivity scores of the selected ligands were predicted using the Molinspiration virtual screening engine tool. Based on the scores, the bioactivity of the ligand molecules can be divided into three categories of score, such as active (> 0.0), moderate (from −5.0 to 0.0) and inactive (< −5.0). The ligands exhibited significant (active and moderate) protease and enzyme inhibition bioactivity scores. Overall, the results demonstrated that all the ligands selected for this study have good molecular and chemical properties and bioactivity scores, suggesting that these ligands can possibly be made orally bioavailable in humans.

### 2.2. Homology Modeling

Three-dimensional structures of the viral membrane protein, SRBD, Nsp14 and TMPRSS2 are not available. Therefore, the structures of the viral membrane protein, SRBD, Nsp14 and TMPRSS2 were computationally modeled based on the template search-identified protein database identifiers 6VXX, 7KJR.A, 5C8S.B and 7MEQ.1.A for crystallographic coordinates, respectively. The structural quality of the models was evaluated using a Ramachandran plot, the MolProbity score, global model quality estimation (GMQE) and qualitative model energy analysis (QMEAN). The different parameters evaluated suggest that all the models have good quality for downstream analysis (Appendix A). The Ramachandran plot of the psi and phi angles in the generated models is shown in Appendix B. The distribution of torsional angles depicted a conformation with fewer clashes. In addition, a comparison of the built models with a set of non-redundant protein structures in the PDB also confirmed model quality. The overall structural configuration is similar to the template according to atomic coordinates root-mean-square deviations (RMSDs) based on structural superposition. In fact, comparison plots demonstrate that the model quality scores of the individual models are comparable with scores attained for experimental structures of similar size. (Appendix C).

### 2.3. Molecular Docking

#### 2.3.1. Molecular Binding Interactions of the Selected Drugs with Structural Proteins (SRBD, Membrane Protein and Nucleocapsid Phosphoprotein) of SARS-CoV-2

**Figure 2:**
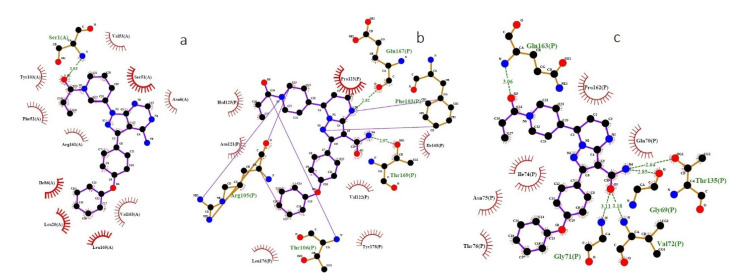
Binding interactions of ibrutinib and zanubrutinib and the structural proteins of SARS-CoV-2 (SRBD, membrane protein and nucleocapsid phosphoprotein) predicted by LigPlot. (**a**) Interaction of the N atom of Ser1 in the SRBD and the O atom of ibrutinib. (**b**) Interaction of the O atom of Glu167 in the membrane protein and the N atom of zanubrutinib. (**c**) Interaction of the O atom of GLY69 and THR135 in the nucleocapsid phosphoprotein and the N atom of zanubrutinib. SRBD—spike protein receptor-binding domain; O—oxygen; N—nitrogen; blue lines—ligand bonds; orange lines—non-ligand bonds; dotted lines—hydrogen bonds and their length; red semicircles—non-ligand residues involved in hydrophobic contacts; black dots—corresponding atoms involved in hydrophobic contacts.

The structural proteins (SRBD, membrane protein and nucleocapsid phosphoprotein) of SARS-CoV-2 were docked with selected ligands. The molecular docking results including the protein–ligand complex binding energy, the number of hydrogen bonds and their distances, and the interacting amino acid residues in the binding cavity of the protein are collected in Table 2. Ibrutinib exhibits the highest-in-magnitude binding energy to the active site residues of SRBD, −7.8 kcal/mol. Moreover, ibrutinib forms a single hydrogen bond (HB) with SER7 (distance of 2.99 Å) of the binding pocket of SRBD (Figure 2a). In contrast, inositol 1,3,4,5-tetrakisphosphate exhibits the lowest binding energy with five hydrogen bonds in the binding pocket of SRBD, −6.5 kcal/mol. Zanubrutinib exhibits the highest binding energy, −8.7 kcal/mol and −7.2 kcal/mol, to the active site residues of nucleocapsid phosphoprotein and membrane protein (Figure 2a,c). Moreover, zanubrutinib forms five hydrogen bonds with the binding pocket of the nucleocapsid phosphoprotein (GLY69, THR135, GLN163, GLY71 and VAL72) and two hydrogen bonds with the binding pocket of the membrane protein (THR169 and GLU167). In contrast, the lowest binding energy of the nucleocapsid phosphoprotein and the membrane protein was observed with inositol 1,3,4,5-tetrakisphosphate.

#### 2.3.2. Molecular Binding Interactions of the Selected Drugs with Nonstructural Proteins (RdRp, Nsp14, Mpro and PLpro) of SARS-CoV-2

**Figure 3:**
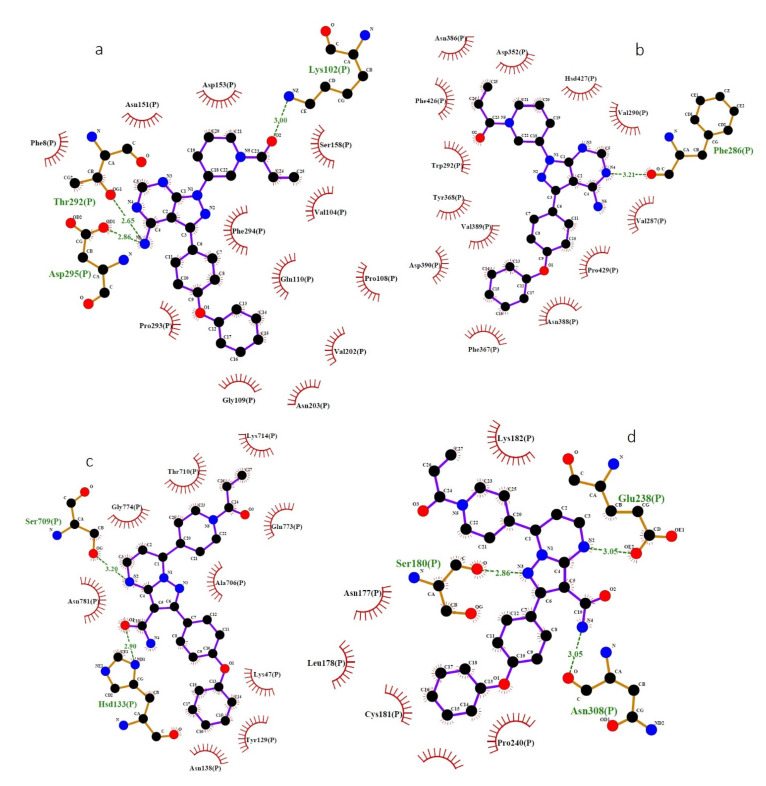
Binding interactions of ibrutinib and zanubrutinib and the nonstructural proteins of SARS-CoV-2 (Mpro, Nsp14, PLpro and RdRp) predicted by LigPlot. (**a**) Interaction of the OG1, OD1, NZ atoms of THR292, ASP295 and LYS102 in Mpro and the N and O atoms of ibrutinib. (**b**) Interaction of the O atom of PHE286 in Nsp14 and the N atom of ibrutinib. (**c**) Interaction of the O, OE2, O atoms of SER180, GLU238, ASN308 in PLpro and the N atom of zanubrutinib. (**d**) Interaction of the ND1, OG atoms of HSD133 and SER709 in RdRp and the O and N atoms of zanubrutinib. Mpro—main protease; RNA-dependent RNA polymerase (RdRp); PLpro—papain-like cysteine protease; blue lines—ligand bonds; orange lines—non-ligand bonds; dotted lines—hydrogen bonds and their length; red semicircles—non-ligand residues involved in hydrophobic contacts; black dots—corresponding atoms involved in hydrophobic contacts.

The molecular docking results for nonstructural proteins (RdRp, Nsp14, Mpro, and PLpro) of SARS-CoV-2 are given in Table 3. Ibrutinib exhibits the highest-in-magnitude binding energy, −8.9 kcal/mol and −8.7 kcal/mol, to the binding pockets of Nsp14 and Mpro, respectively (Figure 3a,b). In addition, ibrutinib forms three hydrogen bonds with the binding pocket of Mpro (THR292 and LYS102) and a single hydrogen bond with the binding pocket of Nsp14 (PHE286). Zanubrutinib exhibits the highest binding energy, −9.0 kcal/mol and −7.3 kcal/mol, to the active site residues of RdRp and PLpro (Figure 3c,d). Moreover, zanubrutinib forms two hydrogen bonds with the binding pocket of RdRp (HSD133 and SER709) and three hydrogen bonds with the binding pocket of PLpro (SER180, GLU238 and ASN308). Further, the lowest complex binding energy of all the selected nonstructural proteins was observed for inositol 1,3,4,5-tetrakisphosphate.

#### 2.3.3. Molecular Binding Interactions of the Selected Drugs with Human ACE2, TMPRSS2 and BTK

**Figure 4:**
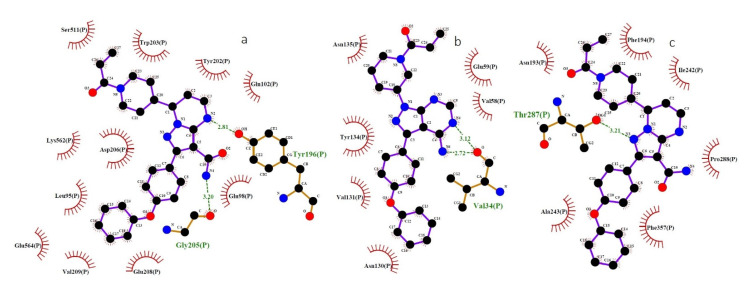
Binding interactions of ibrutinib and zanubrutinib and human proteins (ACE2, BTK and TMPRSS2) predicted by LigPlot. (**a**) Interaction of the OH and O atoms of TYR196 and GLY205 in the ACE2 and the N atom of zanubrutinib. (**b**) Interaction of the O atom of VAL34 in BTK and the N atom of ibrutinib. (**c**) Interaction of the OG1 atom of THR287 in TMPRSS2 and the N atom of zanubrutinib. ACE2—angiotensin-converting enzyme 2; BTK—Bruton’s tyrosine kinase; TMPRSS2—transmembrane serine protease 2; O—oxygen; N—nitrogen; blue lines—ligand bonds; orange lines—non-ligand bonds; dotted lines—hydrogen bonds and their length; red semicircles—non-ligand residues involved in hydrophobic contacts; black dots—corresponding atoms involved in hydrophobic contacts.

Table 4 collects the molecular docking results of human proteins (ACE2, TMPRSS2 and BTK) with the selected ligands. The results indicate that zanubrutinib exhibits the highest-in-magnitude binding energy, −9.8 kcal/mol and −8.2 kcal/mol, to the active site residues of ACE2 and TMPRSS2 (Figure 4a,b). Moreover, zanubrutinib forms two hydrogen bonds with the binding pocket of ACE2 (GLY205 and TYR202) and a single hydrogen bond with the binding pocket of TMPRSS2 (THR287). Accordingly, ibrutinib exhibits the highest binding energy to the binding pocket of BTK, −7.4 kcal/mol (Figure 4c). In addition, ibrutinib forms two hydrogen bonds with the binding pocket of BTK (VAL34). Further, the lowest binding energy of all the selected human proteins (ACE2, TMPRSS2 and BTK) was observed with inositol 1,3,4,5-tetrakisphosphate.

### 2.4. Binding Free Energy

Molecular mechanics-generalized Born surface area (MM/GBSA) calculations were used to efficiently estimate the binding affinities of the ligands for the proteins. The molecular docking results rank the feasible poses of a ligand in the binding pocket based on the scoring function or binding energy (more negative values indicate stronger protein-ligand binding and most stable complexes). MM/GBSA improves the accuracy of molecular docking results and non-redundant binding poses, while yielding approximate binding free energies (more negative values indicate higher binding affinity). According to the results of SBVS, zanubrutinib exhibits the highest-in-magnitude protein-ligand binding energy with ACE2, TMPRS, membrane protein, nucleocapsid phosphoprotein, PLpro and RdRp, while ibrutinib exhibits the highest binding energy with SRBD, Nsp14, Mpro and BTK. Thus, 10 different complexes were selected for XP docking and binding free energy calculations. Table 5 lists the molecular docking score/binding energy and binding free energy of the selected targets with zanubrutinib and ibrutinib obtained with Glide. The highest protein-ligand binding energy of zanubrutinib is obtained for PLpro (−7.0 kcal/mol) and that of ibrutinib for Mpro (−7.8 kcal/mol). Among the structural and nonstructural proteins of SARS-CoV-2, ibrutinib exhibits the highest binding free energy with SRBD (−58.6 kcal/mol) as compared with Nsp14 (−54.4 kcal/mol) and Mpro (−33.2 kcal/mol), while it exhibits a binding free energy of −44.7 kcal/mol with BTK. Further, zanubrutinib exhibits the highest binding free energy with RdRp (−68.3 kcal/mol) as compared with TMPRSS2 (−57.0 kcal/mol), PLpro (−54.3 kcal/mol), Nsp14 (−54.4 kcal/mol), ACE2 (−53.2 kcal/mol), nucleocapsid phosphoprotein (−50.4 kcal/mol) and membrane protein (−50.2 kcal/mol). The ligands that interact the strongest with the protein binding pocket (with highest binding affinity / most negative binding free energies) are selected for further computational studies. Accordingly, the SRBD–ibrutinib, RdRp–zanubrutinib and BTK–ibrutinib complexes are subjected to MD simulations.

### 2.5. MD Simulations

**Figure 5:**
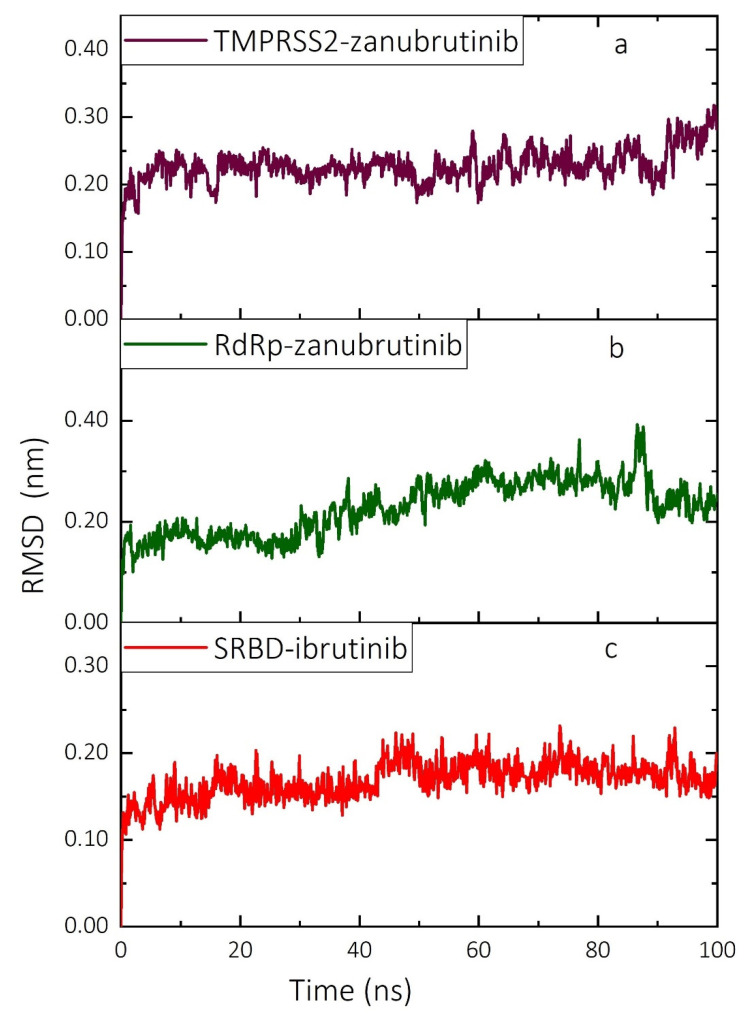
RMSD (nm) of atomic Cα positions for the complexes of targets (SRBD, RdRp and TMPRSS2) and ligands (ibrutinib and zanubrutinib) over the 100-ns timescale of MD simulations performed with NAMD. (**a**) TMPRSS2–zanubrutinib complex, (**b**) RdRp–zanubrutinib complex and (**c**) SRBD–ibrutinib complex. SRBD—spike protein receptor-binding domain; RdRp—RNA-dependent RNA polymerase; TMPRSS2—transmembrane serine protease 2; RMSD—root-mean-square deviation.

**Figure 6:**
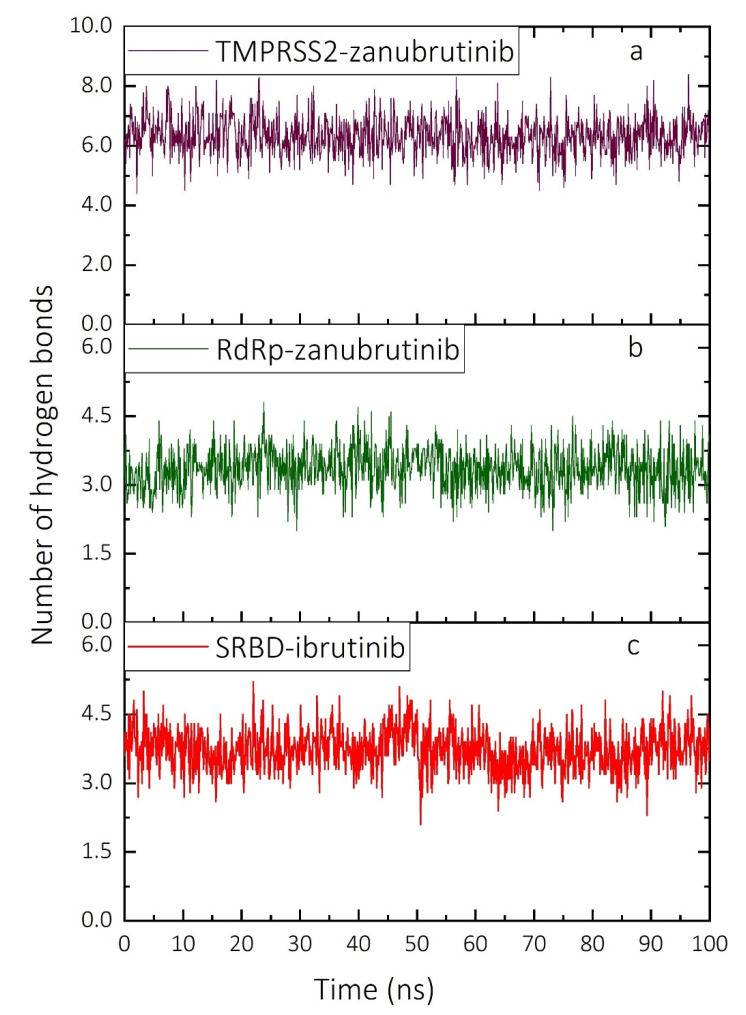
Number of intermolecular HB interactions in the complexes of targets (SRBD, RdRp and TMPRSS2) and ligands (ibrutinib and zanubrutinib) over the 100-ns timescale of MD simulations performed with NAMD. (**a**) TMPRSS2–zanubrutinib complex, (**b**) RdRp–zanubrutinib complex and (**c**) SRBD–ibrutinib complex. SRBD—spike protein receptor-binding domain; RdRp—RNA-dependent RNA polymerase; TMPRSS2—transmembrane serine protease 2; HB—hydrogen bond.

Based on highest-in-magnitude complex binding free energies, MD simulations were performed for the SRBD–ibrutinib, RdRp–zanubrutinib and TMPRS2–zanubrutinib complexes over a 100-ns simulation timescale to test the stability of the complexes in an aqueous environment. MD simulations were performed with the same NAMD protocol, and the atomic Cα position RMSD and the number of hydrogen bonds (HBs) are plotted (for the various frames) along the simulations of the complexes in Figure 5 and Figure 6, respectively. The TMPRSS2–zanubrutinib complex remains quite stable over the simulation timescale, with the complex potentially stabilized due to the active participation of bond-forming amino acid residues in the binding pocket. The mean RMSD value of the TMPRSS2–zanubrutinib complex is relatively small at ~0.23 nm (Figure 5a). Although some structural changes are observed at the N- and C-terminal regions, the active site residues maintain the binding pocket of TMPRSS2 that binds the ligand over the entire simulation timescale. The number of intermolecular HB interactions between TMPRSS2 and the selected ligand complexes (Figure 6a) fluctuates slightly around the average value of ~6.4, reflecting strong stabilizing HB interactions in the system throughout the entire MD simulation timescale.

The RdRp–zanubrutinib complex also remains stable over the MD simulation timescale, with an average RSMD value of ~0.23 nm, even though the RMSD (Figure 5b) exhibits wider fluctuations than for the TMPRS2–zanubrutinib complex. The differences observed in the RMSD values of the protein-ligand complex are due to the binding and unbinding of ligands at various time intervals. The number of intermolecular HB interactions in the RdRp–zanubrutinib complex (Figure 6b) fluctuates around the average value of ~3.4. The SRBD–ibrutinib complex is also stable over the entire 100 ns of simulation with no observed abrupt changes in the RMSD (Figure 5c), which fluctuates around an average value of ~0.17 nm. The fluctuations reflect the dynamic interaction of the close-fitting ligands with amino acid residues in the SRBD binding pocket which stabilize the SRBD–ibrutinib complex. The efficiency of the complex stabilization can be inferred from the average number of HB interactions formed between the SARS-CoV-2 SRBD and ibrutinib, which is ~3.7 (Figure 6c). Moreover, the average number of intermolecular HBs is higher in the RdRp-ligand complex than in the SRBD- and TMPRSS2-ligand complexes.

The HB interactions identified in the molecular docking calculations are preserved in MD simulations, and the average number of HB interactions in MD simulations is more or less equal to that in the molecular docking calculations. In the entire MD simulations of the three different protein-ligand complexes, no evidence was found of significant stacking interactions and the only intermolecular interactions identified were of the HB and hydrophobic type. The relatively small fluctuations in both the atomic Cα position RMSD (Figure 5) and the number of hydrogen bonds (Figure 6) reflect the stability of all target-drug complexes.

## 3. Discussion

**Figure 7:**
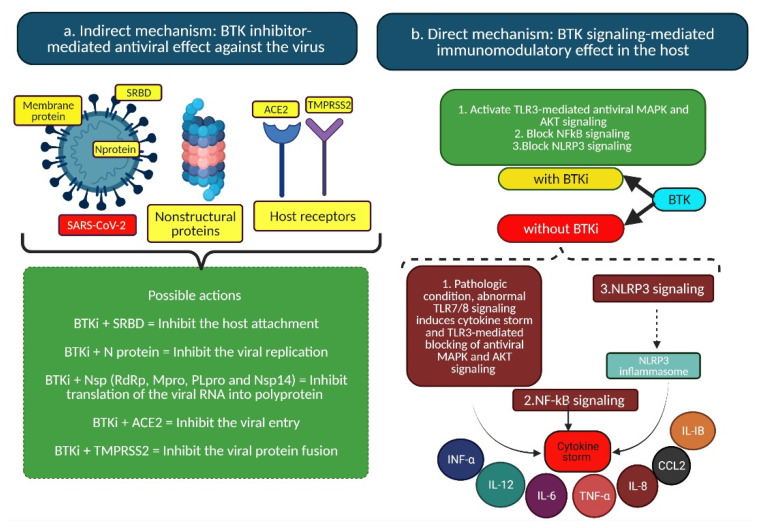
Schematic illustration of the possible mechanism of action of BTK inhibitors: a) direct mechanism of BTKi with BTK signaling-mediated immunomodulatory effect in the host; b) indirect mechanism with BTKi-mediated antiviral effect against SARS-CoV-2. ACE2—angiotensin converting enzyme 2; Akt—protein kinase B; BTKi—Bruton’s tyrosine kinase inhibitor; CCL2—chemokine ligand 2; IL—interleukin; IFN—interferon; MAPK—mitogen-activated protein kinase; NSP—nonstructural protein; NF-κB—nuclear factor-κB; NLRP3—NLR family pyrin domain-containing 3; SRBD—spike protein receptor-binding domain; TLR—toll-like receptors; TMPRSS2—transmembrane serine protease 2; TNF—tumor necrosis factor.

Kinase inhibitors (like BTK) are beneficial regulators of life-threatening symptoms of COVID-19, including anti-inflammatory, cytokine suppression and antifibrotic activity [27,28,29,30,31,32]. Many approved antivirals targeting polymerases or proteases are used to treat emerging viruses, and polymerases and proteases of SARS-CoV-2 were thus selected as drug targets. Moreover, host receptor-targeted inhibitors might regulate the host receptor-dependent virus life cycle, and therefore the viral entry supportive host receptor (ACE2), membrane fusion activator (TMPRSS2) and lung inflammatory mediator (BTK) were also selected as drug targets in this study. Due to their dual (i.e., direct and indirect) action mechanism illustrated in Figure 7, BTK inhibitors such as zanubrutinib and ibrutinib, which exhibit the highest binding affinity to the different structural and nonstructural proteins of SARS-CoV-2 and BTK, are speculated to be potential candidate drugs against SARS-CoV-2 and for cancer patients infected with SARS-CoV-2. Many ongoing clinical studies report that ibrutinib and zanubrutinib are able to reduce COVID-19 disease severity, symptoms and the level of inflammatory cytokines [5,7,33], which is consistent with the molecular docking results of this study.

This computational study proposes two different pathways (indirect and direct mechanism) that possibly regulate the antiviral and immunological effects in the SARS-CoV-2-infected host, including the BTK inhibitor-mediated antiviral response while binding to viral targets and the BTK signaling-mediated immunomodulatory pathway in the host (Figure 7). In general, BTK signaling regulates downstream B cell receptor, Fc receptor and TLR7/8/3 signaling pathways. The inhibition of BTK could elevate the type I interferon levels and activate TLR signaling in systemic lupus erythematosus and different experimental models [34,35,36,37]. Another study reported that blocking the BTK, TLR3-induced protein kinase B (Akt), mitogen-activated protein kinase (MAPK) and nuclear factor (NF)-κB signaling pathways enhanced the antiviral responses in mice models [38]. The aforementioned scientific reports demonstrated that the selected host target BTK possibly regulates the immunomodulatory antiviral responses in the host. Moreover, the binding interaction of off-targets (ACE2 and TMPRSS2) may inhibit viral attachments and viral protein fusion in the host cell. In addition, the BTK inhibitor ibrutinib was reported to reduce the viral titer in Epstein–Barr virus (EBV)-, HIV-infected animal models [39,40] and have immunomodulatory effects on the influenza A virus (IAV)-linked acute lung injury [41]. These reports are consistent with the computational results of this study, including the interaction of BTK inhibitors in the binding cavities of the selected viral targets (i.e., SRBD, membrane protein, nucleocapsid phosphoprotein, RdRp, Mpro and PLpro) that could possibly inhibit viral entry, preprocessing of RdRp and replication.

Ibrutinib exhibits the highest binding affinity for the spike receptor-binding domain. However, it also exhibits considerable binding affinity with human surface receptors ACE2 and TMPRSS2, which undescores the possible role in viral entry into the host cell and inhibition of TMPRSS2-induced membrane fusion, which is required for SARS-CoV-2 replication [42]. In addition, ibrutinib efficiently binds Mpro and Nsp14 of SARS-CoV-2, and it may therefore play an essential role in preventing viral replication and posttranslational processing of viral polyproteins. Moreover, Nsp14 acts as a proofreading exoribonuclease and plays an essential role in viral RNA capping by its methyltransferase activity [43].

Zanubrutinib exhibits the highest binding affinity to the human cell surface receptors ACE2 and TMPRSS2. The activation of the spike protein by TMPRSS2 can make cathepsin activity and lower pH unnecessary for the viral protein to fuse with the host endosomal membrane [42]. The molecular docking results for the TMPRSS2–zanubrutinib complex suggest an important role of zanubrutinib in inhibiting the viral entry into the host cell, probably by increasing the endosomal pH. Moreover, the highest binding affinity of zanubrutinib for the nucleocapsid protein, RdRp and the membrane protein of SARS-CoV-2 supports its inhibitory role in viral replication, nuclear transport and assembly, as membrane protein interaction with the spike and the nucleocapsid protein is required for viral component assembly in the host cell [44]. The binding affinity of both ibrutinib and zanubrutinib for BTK, Mpro and PLpro indicates that both drugs could act as a potential tyrosine kinase and a protease inhibitor as well.

The binding affinities of acalabrutinib, dasatinib, evobrutinib, fostamatinib, inositol, spebrutinib and XL418 were found to be lower than those of ibrutinib and zanubrutinib for all the selected targets. However, those drugs also have a considerable binding affinity with SRBD, membrane protein, nucleocapsid phosphoprotein, RdRp, Nsp14, Mpro and PLpro and with human ACE2, TMPRSS2 and BTK. Inositol 1,3,4,5-tetrakisphosphate exhibits the lowest binding affinity for (and number of hydrogen bonds to) the selected receptors compared to all the selected drugs.

## 4. Materials and Methods

### 4.1. Molecular and Bioactivity Analysis

The cheminformatics tool Molinspiration property engine v2018.10 (https://www.molinspiration.com/ accessed on 3 March 2021), was used to calculate the various molecular properties of the drug candidates. The molecular properties included logP3, topological polar surface area [45], number of atoms, molecular weight, number of HB acceptors and donors, number of rule violations and number of rotatable bonds of the selected ligands [26]. Furthermore, the MiScreen and Molinspiration virtual screening engine v2018.08 were used to predict the biological activity of the given ligands quickly (i.e., 100,000 molecules screened within 30 min) and efficiently. The engine was developed to analyze the binding affinity of ligands to kinase, protease and enzyme inhibitors [46].

### 4.2. Protein and Ligand Preparation

The crystal structure of the selected targets, namely, SARS-CoV-2 Mpro (PDB ID: 6Y2E), PLpro (PDB ID: 6W9C), RdRp (PDB ID: 6M71), nucleocapsid protein (PDB ID: 6VYO), human ACE2 (PDB ID: 1R42) and BTK (PDB ID: 1BTK) were retrieved from the Protein Data Bank (PDB). The amino acid partial charges and the correctness of bond orders, missing atoms and side chains were accounted for using the CHARMM-GUI web interface (https://www.charmm-gui.org/ accessed on 5 March 2021) [47], and all the target structures were cleaned up by removing hetero-atoms and water molecules.

To the best of our knowledge, the 3D structures of the viral membrane protein (Uniprot ID: P0DTC5), SRBD (Uniprot ID: P0DTC2), Nsp14 (Uniprot ID: P0DTD1) and TMPRSS2 (Uniprot ID: O15393) are not available. Therefore, the FASTA sequences of these proteins were retrieved from the Uniprot database [48], and homology modeling was performed using the SWISS-MODEL server with the default settings to search templates and build model functions [49]. The quality of the homology models was assessed using the SWISS-MODEL quality assessment tool (https://swissmodel.expasy.org/assess accessed on 3 March 2021), and the modeled structures were used for molecular docking studies.

Three-dimensional structures of the nine drugs (i.e., BTK inhibitors) acalabrutinib (DrugBank accession No. DB11703), dasatinib (DrugBank accession No. DB01254), evobrutinib (DrugBank accession No. DB15170), fostamatinib (DrugBank accession No. DB12010), ibrutinib (DrugBank accession No. DB09053), inositol 1,3,4,5-tetrakisphosphate (DrugBank accession No. DB01863), spebrutinib (DrugBank accession No. DB11764), XL418 (DrugBank accession No. DB05204) and zanubrutinib (DrugBank accession No. DB15035) were retrieved from the DrugBank database (https://go.drugbank.com/ accessed on 1 March 2021). The structure refinement process involving ligand interconversion and energy minimization was performed using PyRx v0.8 (The Scripps Research Institute, La Jolla, CA, USA). Then, the structures of the selected drugs obtained in structure data format (sdf) were energy-minimized with the universal force field (UFF) and the structures of the ligands were further converted into an AutoDock-compatible file format (.pdbqt) using OpenBabel (Pittsburgh, PA, USA) [50]. The conversion included non-polar hydrogen merged with carbons and polar hydrogen; Gasteiger partial charges were added, and the internal degrees of freedom and torsion were set to zero.

### 4.3. Structure-Based Virtual Screening

Virtual screening of the selected drugs against the SARS-CoV-2 viral proteins SRBD, membrane protein, nucleocapsid phosphoprotein, RdRp, Nsp14, Mpro and PLpro and the human ACE2, TMPRSS2 and BTK was performed using AutoDock Vina in the PyRx software [51] using a Windows 10 Enterprise-supported HP system [Intel(R) Core(TM) i7-8700 CPU@3.20GHz processor with 64-bit operating system and 16GB memory]. Using well-organized gradient-based optimization, scoring functions and multithreading, AutoDock Vina is an efficient program for molecular docking with an improved speed and accuracy (of 78%) over AutoDock 4.0 [52]. Moreover, AutoDock Vina was shown to implement the best scoring functions for both top-scoring and best poses compared to AutoDock, LeDock, rDock, DOCK, LigandFit, Glide, GOLD, Molecular Operating Environment (MOE) and Surflex-Dock [53]. Thus, AutoDock Vina was selected to perform molecular docking. The prepared structure of the protein (.pdb) was provided as a macromolecule and converted into the .pdbqt format. All the prepared drugs (i.e., ligands) were targeted against the selected viral and host proteins in a blind docking manner. The search was performed with the Lamarckian genetic algorithm and an empirical free energy scoring function. The targets and drugs were prepared and molecular docking performed inside a grid box (X-, Y- and Z-axes), with dimensions adjusted to ACE2 (92.71 Å × 71.21 Å × 55.64 Å), BTK (44.95 Å × 47.46 Å × 52.76 Å), membrane protein (39.41 Å × 31.94 Å × 32.97 Å), Mpro (43.18 Å × 69.46 Å × 48.74 Å), nucleocapsid phosphoprotein (48.21 Å × 44.42 Å × 37.84 Å), Nsp14 (99.41 Å × 96.57 Å × 84.46 Å), PLpro (74.81 Å × 54.62 Å × 25.12 Å), RdRp (90.21 Å × 94.05 Å × 69.87 Å), SRBD (54.24 Å × 55.86 Å × 25.12 Å) and TMPRSS2 (83.81 Å × 66.59 Å × 62.46 Å). The results of the analysis were determined by sorting the different protein–ligand complexes with respect to predicted binding energy. Subsequently, the docked complexes were clustered and compared by best-ranked ligand with a RMSD value of 1.0 Å. In total, eight different poses were generated for each ligand and the lowest-energy poses were selected for further analysis. Details of binding energies of different poses of each target–drug complex are given in Appendix D. The atomic interactions of the docked complexes were visualized using LigPlot tools (The European Molecular Biology Laboratory, Hinxton, Cambridgeshire, UK) [54].

### 4.4. Calculation of Binding Free Energy

The drug exhibiting the highest-in-magnitude binding energy with the targets was selected further for XP precision docking [55]. The selected proteins were subjected to the sitemap in Maestro, where random sites were ranked based on the on-site score and the top-ranking site score was used for grid generation. The binding free energy was calculated using the Prime MM/GBSA algorithm with the OPLS-AA force field and the generalized Born/surface area (GB/SA) continuum model. Prime employs a surface generalized Born model using a Gaussian surface instead of a van der Waals surface to better mimic the solvent-accessible surface area [56,57]. The binding free energy was calculated using the following formula

ΔG_bind_ = Δ_E_ + ΔG_solv_ + ΔG_SA_ (1)

Δ_E_ = E_target–ligand complex_ − E_target_ − E_ligand_ (2)

where E_complex_, E_target_ and E_ligand_ are the minimized energies of the target–ligand complex, receptor and ligand, respectively; the solvation free energy difference is evaluated as:

ΔG_solv_ = G_solv(target–ligand complex)_ − G_solv(target)_ − G_solv(ligand)_ (3)

where G_solv(complex)_, G_solv(target)_ and G_solv(ligand)_ are the solvation free energies of the target–ligand complex, target and ligand, respectively; and the non-electrostatic contribution to the solvation free energy is estimated as:

ΔG_SA_ = G_SA(target–ligand complex)_ − G_SA(target)_ − G_SA(ligand)_ (4)

where G_SA(target–ligand complex)_, G_SA(target)_ and G_SA(ligand)_ are the free energies associated with the solvent-accessible surface area for the target–ligand complex, target and ligand, respectively. The Glide docking score (binding energy) and free energy were determined with Schrödinger Release 2020-3 (Schrödinger, LLC, New York, NY, USA, 2020). The top final poses of the target–drug complexes ranked based on the Glide docking scores and Prime MM/GBSA binding affinities were subjected to MD simulations.

### 4.5. Solution Builder and Molecular Dynamics Simulation

The complexes with the highest Glide docking score and binding affinity were used to prepare input files for molecular dynamics (MD) simulations with the CHARMM36 force field [58,59]. The Solution builder plugin of the CHARMM-GUI web interface was employed to prepare solvated complexes with TIP3P water molecules and overall charge-neutral systems with potassium chloride (KCl) ions at a 0.15 mol^−1^ concentration. The initial location of KCl ions was estimated using short Monte Carlo simulations (2000 steps) based on Coulomb and Van der Waals interactions. MD simulations of the solvated complexes were then performed with the nanoscale molecular dynamics (NAMD) program [60]. Long-range Coulomb interactions were determined by the particle mesh Ewald (PME) method [61]. The integrator time step was set to 2 fs, and the simulations were carried out at constant pressure and temperature of 1 bar and 298 K, respectively, using Langevin dynamics with a damping coefficient of 1 ps^−1^. Following 9 × 10^7^ steps of straight energy minimization, the TIP3P water molecules and ions were equilibrated for 2 ns around the rigid proteins (with harmonic restraints). The simulations of the target–ligand complexes were then started from the last frame of restrained equilibration and production runs were performed for up to 100 ns for each complex. Finally, the simulation results, atomic Cα position root-mean-square-deviations (RMSD) and number of hydrogen-bond (HB) interactions, for all drug-target complexes were analyzed with the Visual Molecular Dynamics (VMD) [62] molecular visualization program.

## 5. Conclusions

In conclusion, both ibrutinib and zanubrutinib could be repurposed as potential drugs for the treatment of people affected by COVID-19. Ibrutinib properly binds to the viral SRBD as well as to host surface receptors ACE2, TMPRSS2 and BTK. Thus, it might act as an inhibitor of viral entry into the host cell as well as BTK-mediated inflammatory cytokine storm. Ibrutinib and zanubrutinib also bind to the membrane protein, nucleocapsid protein, RdRp, Nsp14, Mpro and PLpro of SARS-CoV-2. Thus, these two drugs might play an essential role in viral protein assembly, replication and posttranslational processing of viral polyproteins. The results of clinical trials of ibrutinib are promising, and similar studies are recommended for zanubrutinib. Both candidate drugs exhibit a dual action mechanism in the inhibition of the BTK-mediated inflammatory cytokine storm, and possible inhibition of structural and non-structural proteins of SARS-CoV-2 preventing viral entry, assembly, fusion and replication.

## Figures and Tables

**Table 1:** Molecular properties and bioactivity scores of the selected ligands.

Name	DrugBank ID	Mol. wt	LogP ^a^	TPSA (Å^2^) ^a^	nON ^a^	nOHNH ^a^	nviol ^a^	nrotb ^a^	natom ^a^	Bioactivity Score ^b^
Acalabrutinib	DB11703	465.52	2.26	118.52	9	3	0	4	35	KI (0.62); PI (0.17); EI (0.05)
Dasatinib	DB01254	488.06	3.13	106.5	9	3	0	7	33	KI (0.51); PI (−0.27); EI (0.03)
Evobrutinib	DB15170	429.52	4.06	93.38	7	3	0	7	32	KI (0.45); PI (0.19); EI (0.22)
Fostamatinib	DB12010	580.47	2.62	186.74	15	4	2	10	40	KI (0.66); PI (0.28); EI (0.44)
Ibrutinib	DB09053	440.51	3.50	99.18	8	2	0	5	33	KI (0.48); PI (−0.23); EI (0.22)
Inositol 1,3,4,5-tetrakisphosphate	DB01863	500.07	−5.07	307.50	18	10	3	8	28	KI (0.49); PI (0.48); EI (0.70)
Spebrutinib	DB11764	423.45	4.50	97.40	8	3	0	10	31	KI (0.49); PI (−0.20); EI (−0.04)
XL418	DB05204	609.50	4.35	93.28	9	2	1	10	39	KI (0.22); PI (−0.21); EI (0.18)
Zanubrutinib	DB15035	471.56	3.43	102.49	8	3	0	6	35	KI (0.28); PI (−0.04); EI (−0.11)

^a^ Determined by the Molinspiration property engine; ^b^ virtual screening engine tool. KI—kinase inhibitor; PI—protease inhibitor; EI—enzyme inhibitor; nON—number of hydrogen bond acceptors; nOHNH—number of hydrogen bond donors; nviol—number of rule violations; nrotb—number of rotatable bonds; TPSA—topological polar surface area.

**Table 2:** Molecular binding interactions of the selected drugs with the structural proteins (SRBD, membrane protein and nucleocapsid phosphoprotein) of SARS-CoV-2.

Ligand	Binding Energy (kcal/mol)	No. of HBs	Interacting Residues with HBs	HB Distance (Å)
SRBD				
Acalabrutinib	−7.1	2	GLU117, ASP119	3.11, 2.84
Dasatinib	−7.2	1	TRP5	2.70
Evobrutinib	−6.8	1	PRO115	2.94
Fostamatinib	−7.0	4	ILE124, GLU123, ARG106, SER121	2.87, 3.15, 3.22, 3.05
Ibrutinib *	−7.8	1	SER7	2.99
Inositol	−5.6	13	SER111, SER121, ASP119, GLU123, ARG106, ARG109, GLU117	2.91, 2.86, 3.24, 2.93, 2.94, 2.85, 3.26, 3.01, 2.80, 2.94, 3.12, 3.25, 3.30
Spebrutinib	−6.5	5	ARG109, ARG106, ILE124, GLU123	3.16, 3.22, 3.09, 2.96, 3.15
XL418	−7.1	1	SER51	3.30
Zanubrutinib	−7.5	0	NA	NA
Nucleocapsid phosphoprotein				
Acalabrutinib	−7.2	1	ASN75	3.01
Dasatinib	−7.4	6	ASP63, LYS127, ASN126, ILE130	3.08, 2.97, 3.24, 2.88, 3.10, 2.81
Evobrutinib	−7.0	3	THR135, GLN163, GLY69	2.79, 3.20, 3.10
Fostamatinib	−7.6	2	GLY164, VAL172	3.06, 2.90
Ibrutinib	−7.4	2	ASN126	2.97, 3.17
Inositol	−4.9	7	ASP103, GLY60, GLN58, ASP63	2.90, 3.17, 3.17, 2.98, 2.99, 2.80, 2.85
Spebrutinib	−6.7	4	PHE66, THR123, GLY124	2.91, 3.01, 3.22, 3.08
XL418	−7.2	3	ASP63, ASN126, TRP132	3.29, 3.16, 3.30
Zanubrutinib *	−8.7	5	GLY69, THR135, GLN163, GLY71, VAL72	2.85, 2.64, 3.06, 2.11, 3.18
Membrane protease				
Acalabrutinib	−6.6	1	THR169	2.99
Dasatinib	−6.7	3	LEU164, TYR178, ASN121	2.86, 3.27, 3.35
Evobrutinib	−5.3	4	ASN121, GLU167, ARG107	2.94, 3.21, 3.11, 2.90
Fostamatinib	−6.0	5	GLU141, ALA152, ARG150	2.68, 2.88, 2.82, 2.93, 2.92
Ibrutinib	−7.0	0	NA	NA
Inositol	−4.6	8	THR169, GLU167, TYR178, ARG107, ASN121, ARG105	2.88, 3.06, 2.84, 2.81, 3.28, 2.93, 2.89, 3.08
Spebrutinib	−5.6	1	LEU176	3.01
XL418	−6.3	2	THR169, ARG107	3.18, 3.34
Zanubrutinib *	−7.2	2	THR169, GLU167	2.97, 2.82

* indicates the complex selected for further studies; HBs—hydrogen bonds; NA—not available; SRBD—spike protein receptor-binding domain.

**Table 3:** Molecular binding interactions of the selected drugs with nonstructural proteins (RdRp, Nsp14, Mpro and PLpro) of SARS-CoV-2.

Ligand	Binding Energy (kcal/mol)	No. of HBs	Interacting Residues with HBs	HB Distance (Å)
RdRp				
Acalabrutinib	−8.3	1	HSD133	2.78
Dasatinib	−8.5	3	TYR728, GLU58, ASP36	2.96, 3.21, 2.97
Evobrutinib	−7.6	2	THR760, SER772	3.17, 3.33
Fostamatinib	−8.8	7	THR129, ASN781, SER709, LYS47, HSD133	2.69, 2.62, 2.96, 2.82, 2.85, 3.10, 2.82
Ibrutinib	−9.0	3	GLU58, ARG55, ARG733	3.03, 3.17, 2.88
Inositol	−6.9	10	HSD133, SER789, LYS788, ALA706, ASN781, GLY774, TYR129, LYS47	2.70, 3.35, 3.16, 3.19, 2.79, 2.87, 2.96, 2.90, 2.74, 3.04
Spebrutinib	−7.8	4	TYR129, ASN138, LYS47, ASP711	3.19, 3.17, 3.07, 3.11
XL418	−8.6	1	ASN781	3.27
Zanubrutinib *	−9.1	2	HSD133, SER709	2.90, 3.20
NSP14				
Acalabrutinib	−7.5	2	ARG76, TRP247	3.21, 2.96
Dasatinib	−8.1	6	CYS414, GLY416, GLY417, ASP415, VAL287	3.24, 3.21, 2.80, 2.84, 3.07, 2.91
Evobrutinib	−8.3	2	ASP352, ARG400	2.79, 2.72
Fostamatinib	−8.5	5	ASP10, SER28, GLY17,THR5	2.76, 2.92, 2.97, 2.88, 3.19
Ibrutinib *	−8.9	1	PHE286	3.21
Inositol	−5.8	6	PHE73, MET62, GLN246, ILE74, ASP243	3.08, 3.22, 2.62, 2.81, 2.70, 3.13
Spebrutinib	−7.4	2	GLN145, GLU191	3.10, 3.25, 3.22
XL418	−10.5	0	NA	NA
Zanubrutinib	−8.5	3	GLY17, SER28, ASP10	2.80, 2.87, 3.18
Mpro				
Acalabrutinib	−8.5	0	NA	NA
Dasatinib	−7.5	1	ASP245	2.82
Evobrutinib	−7.6	4	ARG40, GLY183, PRO184	3.03, 3.35, 3.17, 3.23
Fostamatinib	−7.4	4	THR111, GLN110	2.80, 3.14, 3.09, 2.80
Ibrutinib *	−8.7	3	THR292, LYS102	2.65, 2.86, 3.00
Inositol	−6.3	14	ASP289, LEU287, TYR239, THR199, ASN238, THR198, ASP238, ARG131	2.79, 3.07, 3.06, 3.00, 3.10, 2.95, 2.71, 3.18, 3.17, 2.95, 3.24, 2.91, 2.90, 3.17
Spebrutinib	−6.9	0	NA	NA
XL418	−8.2	1	GLN110	3.12
Zanubrutinib	−8.1	0	NA	NA
PLpro				
Acalabrutinib	−6.6	1	MET206	2.85
Dasatinib	−6.7	3	ALA176, LEU178, PHE173	2.98, 3.14, 3.10
Evobrutinib	−6.2	2	GLU238, SER180	2.96, 3.15
Fostamatinib	−6.2	2	ASP164, GLY163	2.85, 3.35
Ibrutinib	−7	1	LYS157	3.22
Inositol	−5.7	6	ASN308, SER180, GLU124, LYS126, ASN172	2.96, 2.96, 3.04, 3.10, 3.14, 3.22
Spebrutinib	−6.1	1	ARG166	3.08
XL418	−6.9	2	ASP179, GLN174	2.86, 3.29
Zanubrutinib *	−7.3	3	SER180, GLU238, ASN308	2.86, 3.05, 3.05

* indicates the complex selected for further studies; HBs—hydrogen bonds; Mpro—main protease; NA—not available; RdRp—RNA-dependent RNA polymerase; PLpro—papain-like cysteine protease.

**Table 4:** Molecular binding interactions of the selected drugs with human ACE2, BTK and TMPRSS2.

Ligand	Binding Energy (kcal/mol)	No. of HBs	Interacting Residues with HBs	HB Distance (Å)
ACE2				
Acalabrutinib	−9.4	2	GLN98, TYR196	3.27, 2.85
Dasatinib	−8.9	4	ALA348, ASP382, TYR385	2.98, 2.95, 3.01, 3.06
Evobrutinib	−8.9	1	TYR385	2.81
Fostamatinib	−8.6	2	ASN210, GLU208	2.74, 3.14
Ibrutinib	−9.4	0	NA	NA
Inositol	−6.2	6	GLU208, GLN98, GLN102, TYR202, TYR196	2.70, 2.90, 2.77, 2.71, 2.89, 2.95
Spebrutinib	−8.2	3	ASP382, HSD401, TYR385	2.81, 3.71, 3.12
XL418	−9.2	2	SER43, ASP382	2.80, 3.24
Zanubrutinib *	−9.8	2	GLY205, TYR202	3.20, 2.81
BTK				
Acalabrutinib	−7.3	0	NA	NA
Dasatinib	−6.9	4	ASP107, ASN161, LYS160	3.04, 3.03, 3.04, 2.91
Evobrutinib	−7.3	3	GLU45, PHE44, TYR42	3.30, 3.03, 3.15
Fostamatinib	−6.8	2	ASN170	2.88, 3.09
Ibrutinib *	−7.4	2	VAL34	2.72, 3.12
Inositol	−5.0	4	SER86, GLU90, GLU96	2.94, 2.96, 2.84, 3.14
Spebrutinib	−6.7	2	GLU45, TYR42	3.20, 3.28
XL418	−7.0	0	LYS71	2.70
Zanubrutinib	−7.6	0	NA	NA
TMPRSS2				
Acalabrutinib	−7.4	1	ASN303	3.12
Dasatinib	−8.1	5	GLU385, ASP440, GLY383, CYS465, ASN433	3.06, 3.15, 2.97, 3.31, 3.03
Evobrutinib	−7.4	3	SER441, SER436, GLY462	3.00, 3.24, 3.02
Fostamatinib	−8.1	5	GLY464, GLY439, SER460, VAL280, HSD296	3.15, 3.26, 3.03, 2.88, 3.09
Ibrutinib	−7.6	1	ASN192	3.10
Inositol	−5.4	10	LEU378, GLY377, ASN451, GLN253, ASP144, SER448, ASN450	2.81, 3.12, 2.92, 2.89, 2.92, 2.98, 3.16, 3.07, 2.94, 3.14
Spebrutinib	−6.5	1	ASN192	3.03
XL418	−7.6	3	PHE156, ASN451, CYS241	3.26, 3.15, 2.80
Zanubrutinib *	−8.2	1	THR287	3.21

* indicates the complex selected for further studies; ACE2—angiotensin-converting enzyme 2; BTK—Bruton’s tyrosine kinase; HBs—hydrogen bonds; NA—not available; TMPRSS2—transmembrane serine protease 2.

**Table 5:** Docking score (binding energy) and binding free energy of the selected target zanubrutinib and ibrutinib complexes.

No.	Name of the Complex	Binding Energy (kcal/mol)	ΔG (kcal/mol)
1	ACE2–zanubrutinib	−6.4	−53.2
2	TMPRSS2–zanubrutinib	−5.8	−57.0
3	Membrane protein–zanubrutinib	−5.1	−50.2
4	Nucleocapsid phosphoprotein–zanubrutinib	−6.7	−50.4
5	PLpro–zanubrutinib	−7.0	−54.3
6	RdRp–zanubrutinib	−6.3	−68.3
7	SRBD–ibrutinib	−6.0	−58.6
8	Nsp14–ibrutinib	−6. 6	−54.4
9	Mpro–ibrutinib	−7.8	−33.2
10	BTK–ibrutinib	−5.7	−44.7

ACE2—angiotensin-converting enzyme 2; BTK—Bruton’s tyrosine kinase; Mpro—main protease; Nsp14—nonstructural protein 14; RdRp—RNA-dependent RNA polymerase; PLpro—papain-like cysteine protease; SRBD—spike protein receptor-binding domain; TMPRSS2—transmembrane serine protease 2.

**Figure A1:**
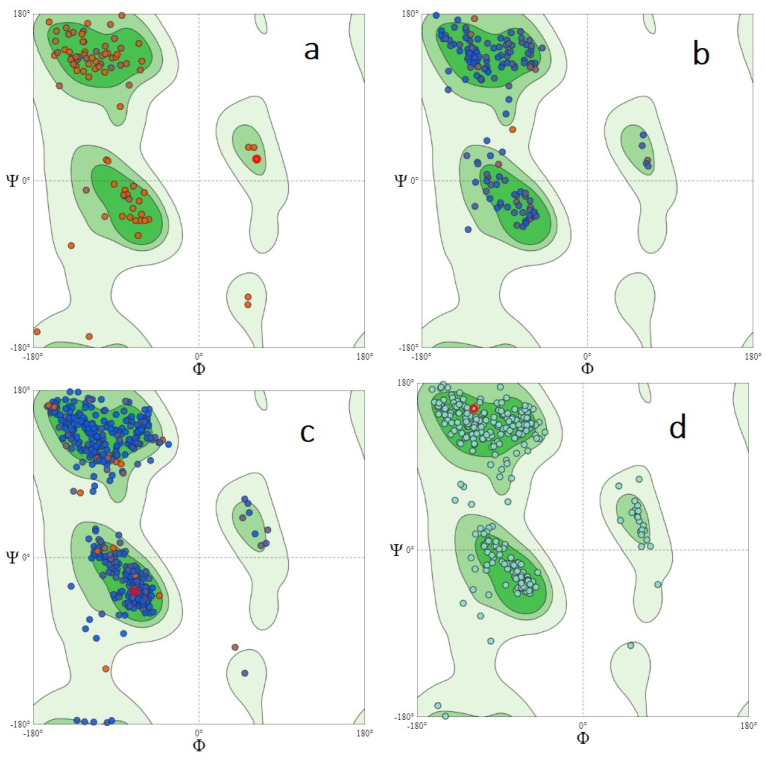
Ramachandran plot of the modeled structures. Ramachandran plot of the modeled structures: (**a**) membrane protease; (**b**) spike receptor-binding domain; (**c**) nonstructural protein 14; (**d**) transmembrane protease, serine 2. 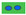 General glycine/pre-proline/proline favoured regions; 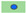 General glycine/pre-proline/proline allowed regions; 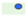 Glycine favoured regions; 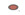 Glycine allowed regions.

**Figure A2:**
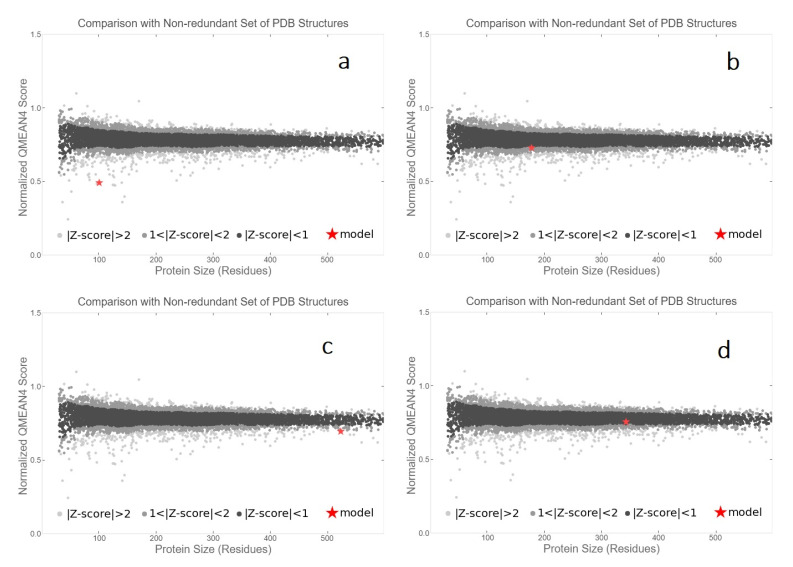
Comparison of the modeled structures to the set of non-redundant crystallographic proteins in the Protein Data Bank: (**a**) membrane protease; (**b**) spike receptor-binding domain; (**c**) nonstructural protein 14; (**d**) transmembrane protease, serine 2. The plots demonstrate that the model quality scores of the individual models are comparable with scores attained for experimental structures of similar size. The x-axis represents the number of residues in the protein; the y-axis represents the QMEAN score. Each dot indicates a single experimental protein structure. Normalized QMEAN scores within 1 standard deviation of mean, Z score (0 to 1), are shown in black dots, those with a Z score between 1 and 2 in grey, and those with a Z score above 2 in light grey, while the actual model is represented as a red star.

## Data Availability

Not applicable.

## Appendix A

**Table A1:** Evaluation of the structural quality of the modeled structures.

Quality Parameters	Membrane Protease	SRBD	Nsp14	TMPRSS2
Template	6VXX	7KJR.A	5C8S.B	7MEQ.1.A
RMSD (Å) from template	0.6	0.8	0.8	
QMEAN	−5.68	−1.12	−2.15	−0.51
GMQE	0.19	0.88	0.93	0.53
MolProbity score	2.21	0.72	2.59	1.24
Ramachandran favoured (%)	92.93	96.59	95.98	95.03
Ramachandran outliers (%)	2.02	0.00	0.38	0.29
Rotamer outliers (%)	0.00	0.00	15.87	0.68
Bad bonds	0/808	1/1454	3/4338	0/2766
Bad angles	17/1094	7/1981	69/5900	20/3766

Notes: Nsp14-non-structural protein 14; SRBD-spike receptor binding domain; TMPRSS2-transmembrane protease, serine 2.

## Appendix D

List of binding poses of each target–drug complex. The binding poses of the target-drug complex have highest in magnitude of binding energy was selected for further analysis. ACE2—angiotensin-converting enzyme 2; BTK—Bruton’s tyrosine kinase; MD—molecular dynamics; Mpro—main protease; nsp—nonstructural protein; PLpro—papain-like protease; RdRp—RNA-dependent RNA polymerase (RdRp); RMSD-root-mean-square deviations; SRBD—spike receptor-binding domain; TMPRSS2—transmembrane serine protease 2.
